# Therapeutic effect of topical administration of red onion extract in a murine model of allergic rhinitis

**DOI:** 10.1038/s41598-019-39379-9

**Published:** 2019-02-27

**Authors:** Min Young Seo, Ki Ryung Kim, Jung Joo Lee, Gwanghui Ryu, Seung Hoon Lee, Sang Duk Hong, Hun-Jong Dhong, Chung-Hwan Baek, Seung-Kyu Chung, Hyo Yeol Kim

**Affiliations:** 1 Department of Otorhinolaryngology - Head and Neck Surgery, Korea University College of Medicine, Korea University Ansan Hospital, Ansan, South Korea; 2 Department of Otorhinolaryngology - Head and Neck Surgery, Samsung Medical Center, Sungkyunkwan University School of Medicine, Seoul, Korea

## Abstract

The aim of this study was to evaluate the effect of topical administration of onion (*Allium cepa)* extract on nasal cavity for treatment of allergic rhinitis (AR). BALB/c mice were sensitized by intraperitoneal injection of ovalbumin (OVA) and challenged with intranasal instillation of OVA with or without onion extracts for five times a week on 3 consecutive weeks. Allergic symptom score according to frequencies of sneezing, serum total and OVA specific immunoglobulin E (IgE) level, cytokine levels of nasal mucosa and eosinophilic infiltration were analyzed. Allergic symptom score, serum total and OVA specific IgE, cytokine levels of nasal mucosa (interleukin (IL)-4, IL-5, IL-10, IL-13, IFN-γ, TNF-α and COX-2) and eosinophilic infiltration were higher in allergic mouse group than negative control group. Topical application of onion extracts significantly reduced allergic symptoms and OVA specific IgE levels. Cytokine levels of IL-4, IL-5, IL-10, IL-13 and IFN-γ were significantly decreased in groups treated with onion extract. In addition, eosinophil infiltration of nasal turbinate mucosa was also significantly decreased after treatment with onion extract. Topical administration of onion extract significantly reduces allergic rhinitis symptom and allergic inflammatory reaction in a murine allergic model. It can be assumed that the topical application of onion extract regulates allergic symptoms by suppressing the type-1 helper (Th1) and type-2 helper (Th2) responses and reducing the allergic inflammatory reaction.

## Introduction

Allergic rhinitis (AR), one of the most common diseases in rhinology clinic, is mediated by immunoglobulin E (IgE) response. In Korea, the prevalence of AR has been estimated to be 18.5% to 28% with a mean annual cost of 18,000 US dollars^1–3^. For treatment of AR, oral H1-antihistamines and intranasal corticosteroids are most commonly used as first treatment. Allergen immunotherapy (AIT) is also an effective treatment by modifying the natural course of AR. However, some patients do not respond to such treatment and half of physicians do not prescribe AIT for AR in our country^4^. Furthermore, it is impossible to apply these therapeutic modalities in patients with special circumstances such as pregnancy, infant, and the elderly. Thus, alternative therapeutic material needs to be developed based on natural extract for AR.

*Allium cepa* (onion) is a widely used medicinal herb for folk remedy to treat upper airway disease in Korea. These days, many people ingest extract from *Allium cepa* for treatment of allergic or upper airway disease in our country. In general, most health physicians regarded it as just an unproven folk remedies. However, some research studies supports its efficacy. Dorsch *et al*.^5^ have reported that onion extract have the anti-asthmatic effect via leukotriene or thromboxane biosynthesis and histamine release inhibition. Thromboxane plays a major role in immediate asthmatic response and leukotrienes also plays a central role in the pathogenesis of asthma and many inflammatory diseases^6,7^. In addition, Kaiser *et al*.^8^ also have reported that onion extract can stabilize the mast cell activity in murine allergic model. Mast cell plays a central role in allergic reaction according to synthesis or release of histamine. Histamine is also asoociated with the inflammatory reaction^9^. Thus, they have suggested that onion extract possessed anti-inflammatory reaction and this could be related to its anti-histaminic reaction. They also have reported that inhibition of eosinophil peroxidase (EPO) activity with onion extract treatment. It is well known that eosinophils have play an important role in allergic airway inflammation and its granular constituent EPO has plays an important role via oxidative stress^10^. Thus, onion extract treatment has the anti-allergic and anti-inflammatory effect via various mechanisms.

However, these studies evaluated the effects of systemic administration of onion extract. To the best of our knowledge, no study has reported the therapeutic effect of topical administration of *Allium cepa* extract. Therefore, the objective of this study was to evaluate the effect of topical nasal administration of *Allium cepa* extract on AR using a murine model.

## Results

### Initial experiment

**Figure 1:**
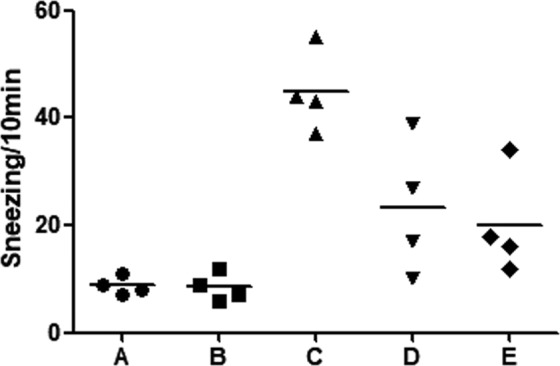
Sneezing symptom scores of initial experiment. *P < 0.05, **P < 0.001.

**Figure 2:**
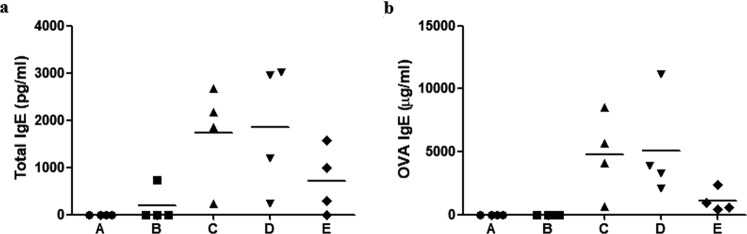
Mean value of total IgE (ng/ml, **a**) and OVA specific IgE (μg/ml, **b**) of initial experiment. *P < 0.05, **P < 0.001.

**Figure 3:**
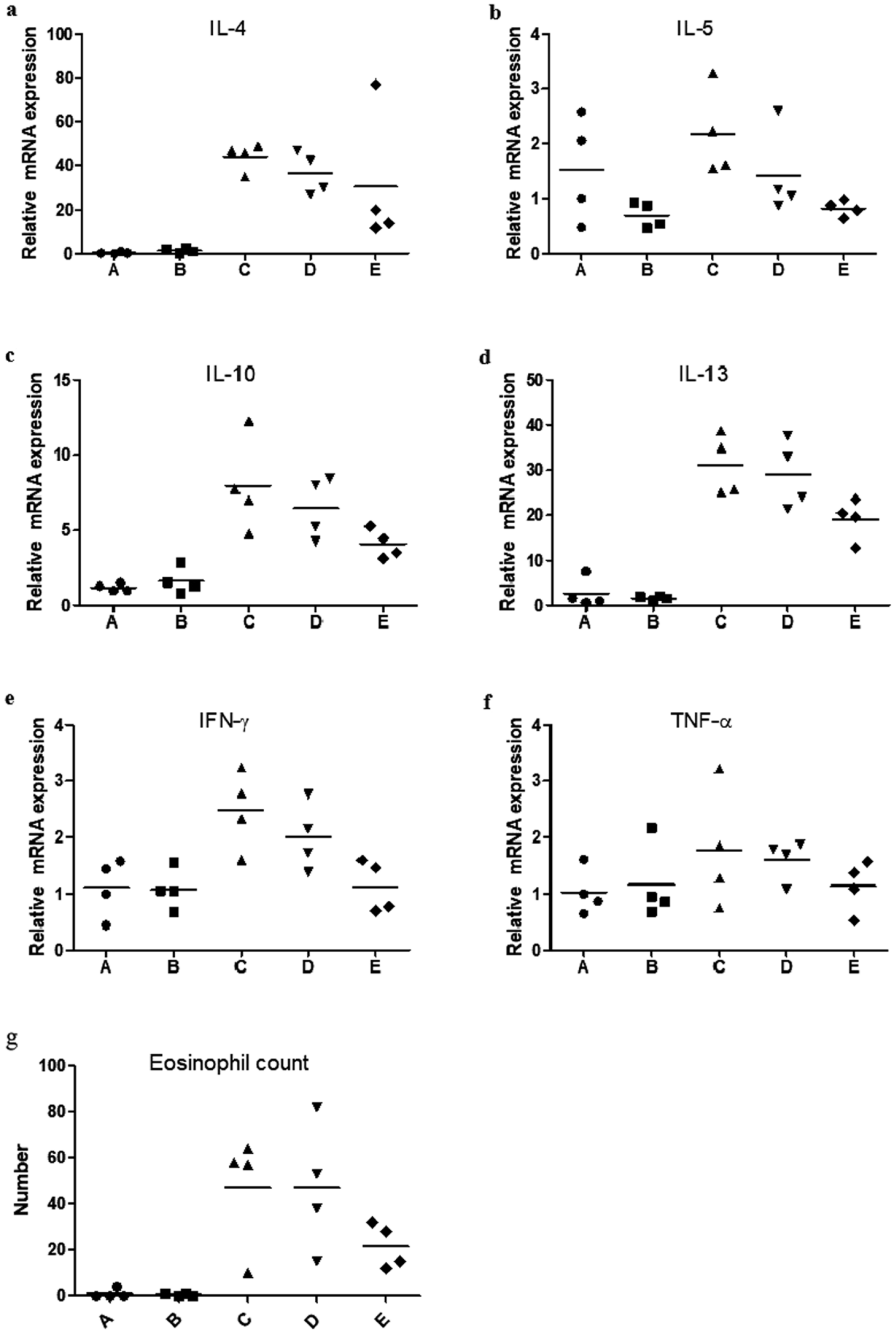
Relative mRNA expression of IL-4 (**a**), IL-5 (**b**), IL-10 (**c**), IL-13 (**d**), IFN-γ (**e**), TNF-α (**f**) and eosinophil count (**g**)  in nasal mucosa of the initial experiment. *P < 0.05, **P < 0.001.

We performed an initial experiment to evaluate the effect of onion extract on normal mice and determined its proper concentration. Figure 1 shows sneezing counts for each group during 10-minute period after intranasal challenge with ovalbumin (OVA). Mice in group C (positive control; OVA/OVA) sneezed more frequently than those in group A (negative control; phosphate-buffered saline (PBS)/PBS) and group B (onion 40 μL exposure group; PBS/PBS + onion extract 40 μL). However, sneezing frequencies were lower in group D (onion 20 μL treatment group; OVA/OVA + onion extract 20 μL) and group E (onion 40 μL treatment group; OVA/OVA + onion extract 40 μL) than group C. Serum total IgE and OVA specific IgE levels were higher in group C than those in groups A and B, but lower in group E. However, we coud not find a decrease in total IgE levels in group D (Fig. 2). According to results of RT-PCR analysis, mRNA levels of cytokines (Interleukin (IL)-4, IL-5, IL-10, IL-13, interferon- γ (IFN-γ) and tumor necrosis factor-α (TNF-α)) were decreased after treatment with onion extract. Such decreases were more prominent in group E than those in group D (Fig. 3). In the initial experiment, there were no significant differences of allergic symptom score, serum IgE levels and mRNA expression of cytokines between groups A and B. Thus, we consider that topical application of onion extracts did not cause an inflammatory reaction in normal mice. Although there was no statistical significance between each group because of small number of mice (n = 4 each), the *p*-value was less than 0.05 in symptom score, IL-5, IL-13 and IFN-γ before Bonferroni’s corrections in the comparison of group C and group E.

### Second experiments

**Figure 4:**
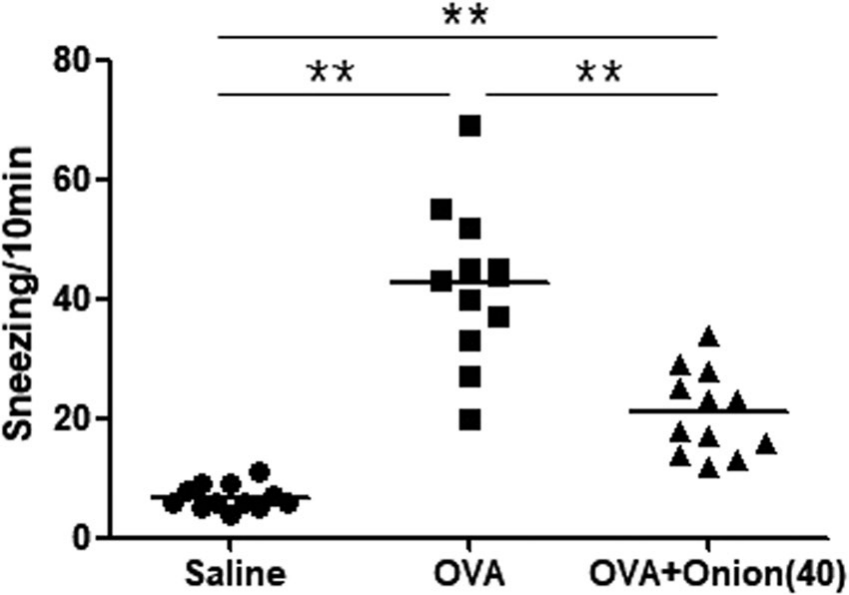
Sneezing symptom scores of final experiment. *P < 0.05, **P < 0.001.

Based on results of the initial experimental, we performed additional experiment by adding 8 mice to groups A, C, and E. Based on sneezing scores, mice in group C sneezed significantly (*P* < 0.001) more frequently than those in group A. Sneezing score in group E was significantly lower than that in group C (*P* < 0.001) (Fig. 4).

**Figure 5:**
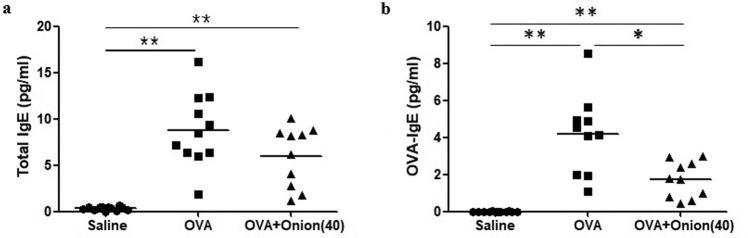
Mean value of total IgE (ng/ml, **a**) and OVA specific IgE (μg/ml, **b**) of final experiment. *P < 0.05, **P < 0.001.

Serum level of total IgE was significantly (*P* < 0.001) higher in group C than that in group A. Total IgE level was not lowered after treatment with onion extract. However, serum OVA specific IgE level was significantly (*P* = 0.01) lower in group E than that in group C (Fig. 5).

**Figure 6:**
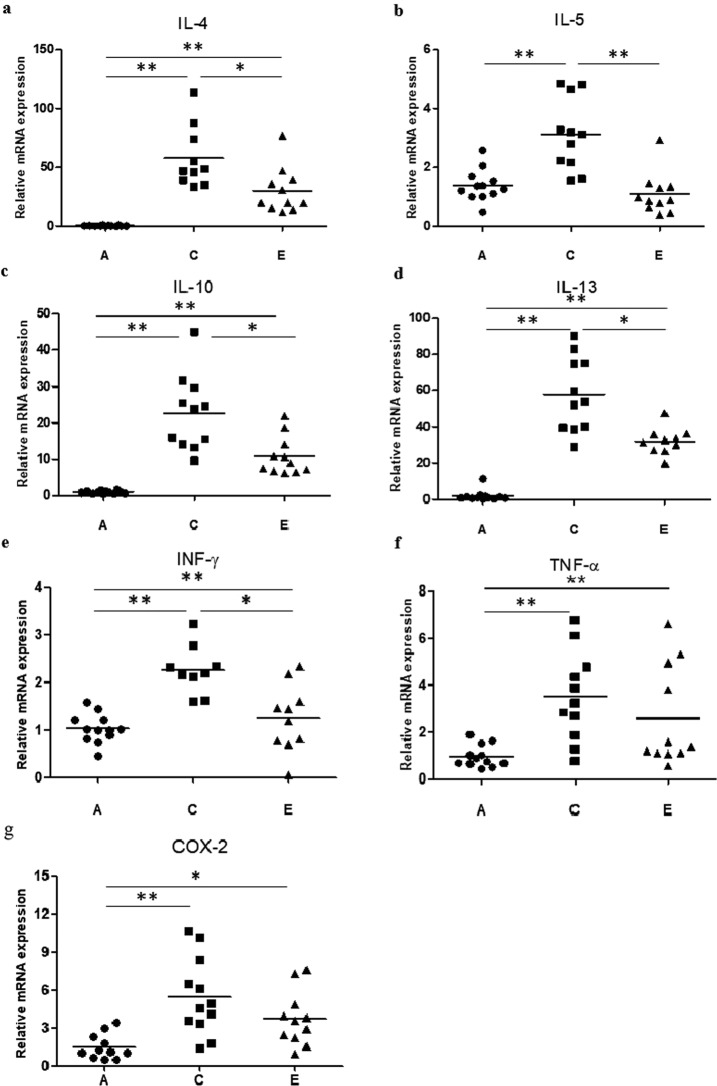
Relative mRNA expression levels of IL4 (**a**), IL-5 (**b**), IL-10 (**c**), IL-13 (**d**), IFN-γ (**e**), TNF-α (**f**) and COX-2 (**g**) in nasal mucosa of the final experiment. *P < 0.05, **P < 0.001.

**Figure 7:**
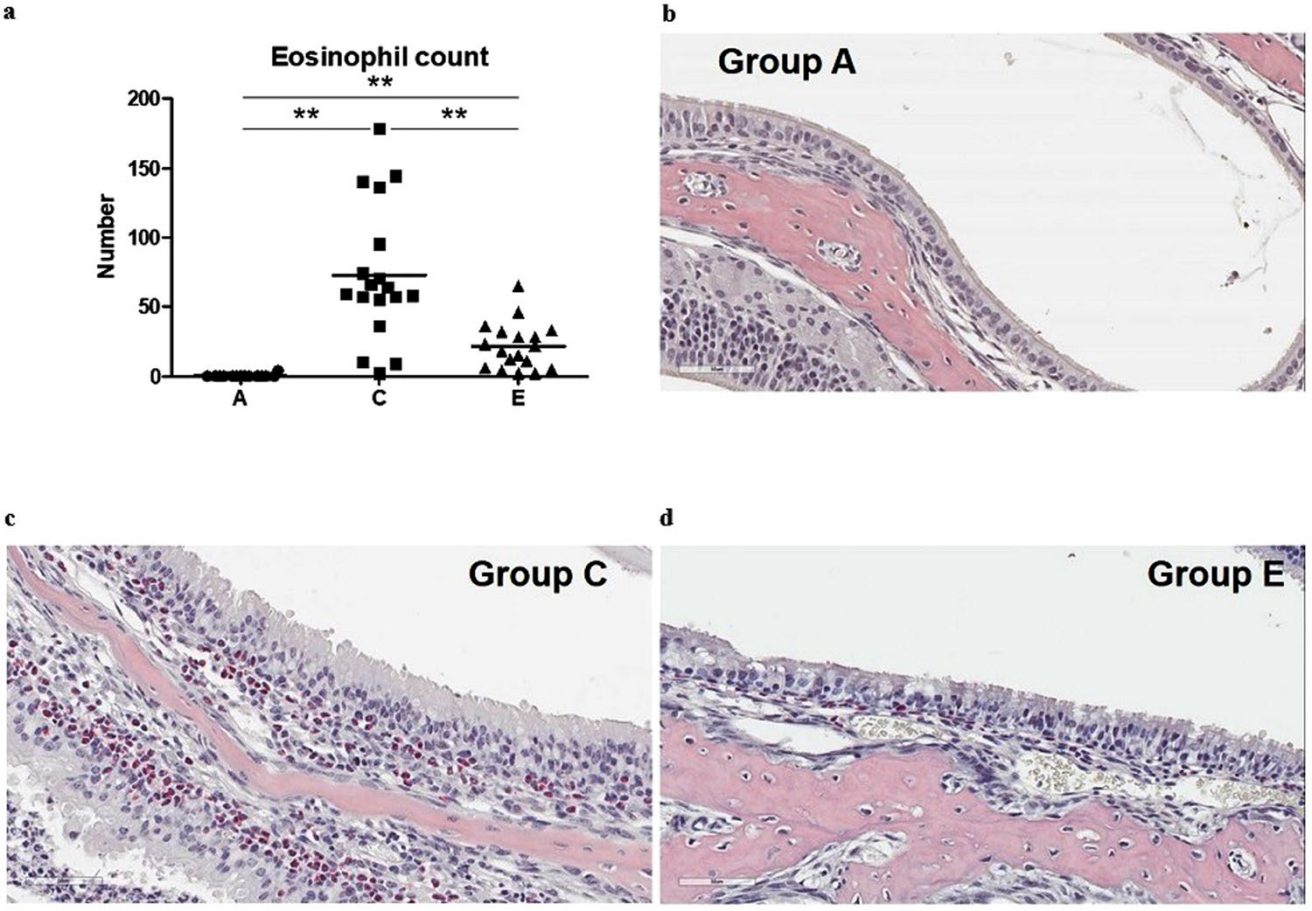
Number of eosinophil infiltration of nasal inferior turbinate mucosa (**a**) and Sirius Red staining (400 x magnification) of each group (**b**) group A, (**c**) group C, (**d**) group E). *P < 0.05, **P < 0.001.

Results of qPCR analysis showed that mRNA levels of IL-4, IL-5, and IL-13 were significantly higher in group C than those in group A (all *P* < 0.001). Their levels were lower in group E than those in group C (*P* = 0.008, *P* < 0.001, and *P* = 0.001, respectively). IL-10 and IFN-γ mRNA levels were also significantly higher in group C than those in group A (both *p* < 0.001). Their levels were also lower in group E than those in group C (*p* = 0.006 and *p* = 0.012, respectively). Although mRNA levels TNF-α and cyclooxygenase-2 (COX-2) were also significantly higher in group C than those in group A (*P* < 0.001, each), their levels in group E were not significantly lower than those in group C (*P* = 0.514, *P* = 0.151, respectively) (Fig. 6). Eosinophil infiltration of nasal turbinate mucosa was significantly (*P* < 0.001) more severe in group C than that in group A. This infiltration was significantly (*P* < 0.001) lower in group E than that in group C (Fig. 7).

## Discussion

IL-4, IL-5, and IL-13 are produced by type-2 helper (Th2) cells that play a major role in the induction and maintenance of allergic inflammatory process. During allergic inflammatory reaction, IL-5 induces differentiation, activation, and survival of eosinophils while IL-4 and 13 promote the production of IgE^11^. Previous studies have shown that systemic administration of onion extract has anti-inflammatory and anti-allergic effects. Oliveira *et al*. have reported that oral administration of onion extract can reduce the production of inflammatory cytokines such as IL-4, IL-5, and IL-13 in a murine allergic model^12^. Kaiser *et al*. have also reported that oral administration of herbal product derived from onion bulb can attenuate type I allergic reaction by its antihistaminic, anti-inflammatory, and antioxidant activities^8^. In the present study, mRNA levels of IL-4, IL-5, and IL-13 were significantly higher in allergic mouse model than those in the negative control group. In addition, their levels were decreased in groups treated with onion extract. We also found that topical application of onion extracts reduced allergic reaction and local inflammation through various parameters such as sneezing score, serum OVA specific IgE, mRNA levels of cytokines by qRT-PCR, and histology analysis of expression of eosinophils.

It is believed that quercetin is the main component of onion extract that is effective in controlling allergic rhinitis. Quercetin is a naturally derived polyphenol and flavonoid and its main sources are vegetables and fruits^13,14^. Many studies have shown that quercetin possesses anti-allergic or anti-inflammatory effects that could be used to treat allergic disease^12,13^. Onion is one of the richest sources of quercetin. The amount of quercetin decreases from dry skin to inner rings of onion^13^. Therefore, we only crushed the outer two layers of onion without removing the skin. Several studies have reported that quercetin possesses anti-inflammatory and anti-allergic effects. Park *et al*.^15^ have reported that quercetin can decrease gene expression and production of pro-inflammatory cytokines from mast cells. In addition, Park *et al*.^16^ have reported that quercetin can reduce Th2 cytokine production. Thornhill *et al*.^17^ have reported that systemic administration of quercetin inhibits inflammatory processes attributed to activated neutrophils, prevention of mast cell and basophil degranulation and inhibition of leukotriene production. In addition, they also have suggested that the recommended dosage from 250–600 mg, three times daily for allergic rhinitis. Furthermore, Hirano *et al*.^18^ and Kawai *et al*.^19^ also have reported that systemic intake of enzymatically modified isoquercitrin relieves the allergic symptoms caused by Japanese cedar pollinosis. Remberg *et al*.^20^ have reported that significant nasal and ocular symptom relief was observed in allergic rhinitis patients with topical nasal application of *Artemisia abrotanum* L. extract, which contains flavonols such as centauredin, casticin and quercetin. Thus, we also thought that topical application of onion extracts might have effects for control of allergic rhinitis symptoms.

In addition to quercetin, other substances contained in onions such as luteolin, fisetin, apigenin and benzyl-isothiocyanates have also been shown to have anti-asthmatic effects^8,21–23^. However, subsequent studies on anti-asthmatic effects of these substances are less common than quercetin. Therefore, it is important to analyze the various substances contained in the onion extract and to confirm its effect for clinical application.

IL-10 plays a key role in the differentiation and function of regulatory T cells^24^. In the present study, mRNA levels of IL-10 was higher in allergy mouse group than that in the negative control group. However, its level was lower in onion extract applied group than that in allergy mouse group. In addition, mRNA level of IFN-γ, which the type-1 helper (Th1) cytokine, was higher in allergy mouse group than that in the negative control group. However, its level was lower in onion extract applied group than that in allergy mouse group. IFN-γ values also showed similar tendency to those of IL-10. IL-10 is a multifunctional cytokine and one of its important roles is related to inflammatory reactions^24^. Considering these results, we concluded that topical application of onion extract does not seem to have regulatory T cell activity. It can be assumed that topical application of onion extract can regulate allergic symptoms by suppressing both Th1 and Th2 responses and reducing allergic inflammatory reaction. For confirmation of local allergic inflammatory reaction, we also checked the COX-2 mRNA levels also. COX-2 is implicated in inflammatory processes and assumed to be dynamically regulated, responding to inflammatory stimuli^25^. Jang *et al*.^26^ reported that COX-2 expression was significantly increased in the allergic mice and inhibition of COX-2 exerts an anti-allergic effect through the downregulation of prostaglandin D2. In present study, COX-2 level was significantly higher in allergic mouse model group than those in the negative control group. Although their levels were not statistically significantly lower in onion extract applied group compared to those in allergic mouse model group, they showed the level tended to be low in onion extract applied group. Therefore, we suggest that topical application of onion extracts might reduce local inflammatory reaction by suppressing both Th1 and Th2 responses.

This study has several limitations. First, we only focused on Th1 and Th2 cytokines. A recent study has shown that type-17 helper (Th17) may also play a crucial role in the pathophysiology of allergic inflammatory upper airway disease^27^. However, the concept of allergic inflammatory diseases are still based on dysregulation of Th1/Th2 balance and Th2-predominant inflammation is important in allergic disease. Second, onion extract used in this study was juice made by grinding raw red onion. Thus, direct application to human nasal mucosa might be limited due to local irritation. Therefore, additional studies should be conducted to extract only quercetin and quantitatively analyze its efficacy in clinical trials.

## Conclusions

In summary, topical administration of onion extract could significantly reduce allergic rhinitis symptom and allergic inflammatory reaction in a murine allergic model. Further research is needed to develop topically applicable onion extracts without causing sinonasal irritation.

## Methods

### Animals

Forty-four healthy female BALB/c mice (6-week-old, weighing 17–18 g each) were purchased from Orient Bio Inc. (Gapyeong, Korea). All animal experiments were performed under specific pathogen free conditions in accordance with Guidelines for Animal Experimentation. Animal experiment protocol was approved by Institutional Animal Care and Use Committee (IACUC) of Samsung Biomedical Research Institute, Seoul, Korea (IACUC number: 20170117001).

### Preparation of red onion extract

To prepare red onion aqueous extract, red onions were purchased from Garak Market (Seoul, Korea). Only the outer two shells including the skin of onion were used. Fresh red onions were ground using Hurom green juicer (Hurom, Seoul, Korea) and centrifuged at 4,000 rpm for 15 minutes at 4 °C. The crude extract was carefully transferred to a new tube and immediately filtered through a 70 µm nylon mesh (BD Bioscience, CA, USA). The mesh was squeezed by hand to remove debris. The final aqueous extract of red onion was stored at −20 °C before use.

### Animal experimental protocols

**Figure 8:**
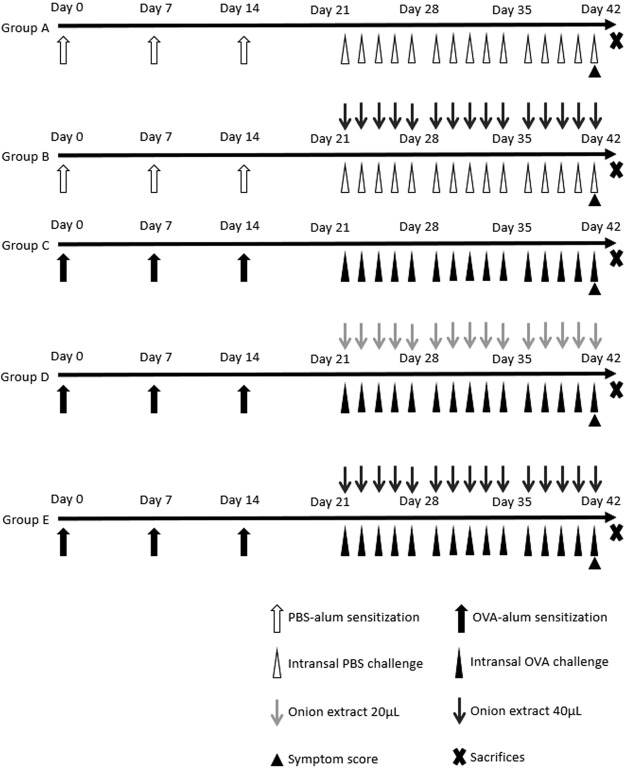
Experimental protocol. PBS: phosphate-buffered saline, OVA: ovalbumin.

For initial assessment, mice were divided into five groups (4 mice per group): (1) group A, PBS/PBS group, (2) group B, PBS/PBS + onion extract 40 μL, (3) group C, OVA/OVA group, (4) group D, OVA/OVA + onion extract 20 μL, and (5) group E, OVA/OVA + onion extract 40 μL. The protocol of allergen sensitization and treatment is summarized in Fig. 8. In allergic model (groups C, D, E), mice were sensitized by intraperitoneal (IP) injection with 25 μg of OVA (grade V; Sigma-Aldrich, St. Louis, MO, USA) and 1 mg of aluminum hydroxide (Sigma-Aldrich) dissolved in 200 μL of PBS on days 0, 7, and 14. These OVA sensitized mice were challenged with intranasal instillation of OVA 100 μg in 20 μL of PBS into bilateral nasal cavities with or without onion extract five times a week for three consecutive weeks from day 21 to day 41. Control mice (group A, B) were treated with PBS instead of OVA and onion extract using the same protocol. At day 41, 15 min after the final intranasal challenge, we recorded behaviors of mice during a 10-minute period to count frequencies of sneezing. Mice were then euthanized and sacrificed at 24 hours following the last intranasal challenge. Nasal tissues and blood were collected for further experiments.

### Histological analysis

Nasal tissues including nasal turbinate and septum were fixed immediately with 4% paraformaldehyde solution (Biosesang, Sungnam, Korea), decalcified with 10% EDTA solution for several days, and embedded in paraffin. Anatomically similar sections of nasal tissues were stained with Sirius Red stain for the detection of eosinophils. This stain protocol was followed as previously described with slight modifications. Briefly, sections were stained with Mayer’s hematoxylin (DAKO, Denmark) for three minutes and rinse in tap water followed by a rinse in absolute ethanol. The sections were placed into alkaline sirius red (Sigma-Aldrich, MO, USA) for 2 hours and rinsed well in tap water. The stained slides were cleared by xylene and mount with permount medium^28^. A single blinded observer counted eosinophils under a light microscope (×400 magnification) at the inferior turbinate mucosa.

### ELISA of Serum IgE

Blood samples were collected by cardiac puncture 24 hours after the last intranasal challenge. Serum was obtained using BD Microstrainer (BD Bioscience, CA, USA) and stored at −20 °C until analyzed. Serum levels of total IgE and OVA-specific IgE were measured using commercially available ELISA kits (mouse IgE ELISA kit, BD Biosciences, CA, USA; mouse OVA-specific IgE, MD bioproducts, Zurich, Switzerland) following manufacturers’ procedures. After completing the assay, optical density (OD) was read immediately at wavelength of 450 nm using an xMark Microplate reader (Bio-Rad, CA, USA).

### Quantitative real-time PCR (qRT-PCR)

Nasal turbinate mucosa tissues were harvested, immediately submerged in RNAlater solution (Ambion, TX, USA), and stored at 4 °C until RNA extraction. Total RNA was extracted from nasal turbinate mucosa tissues using TRIZol reagent (Invitrogen, CA, USA). Purified RNA (1 μg) was then reverse transcribed to cDNA using a SuperScript™ VILO™ cDNA Synthesis Kit (Invitrogen, Carlsbad, CA, USA) according to the manufacturer’s instructions. qRT-PCR was performed with TaqMan method using a 7900HT Fast Real-Time System (Applied Biosystems, CA, USA) with pre-designed primer and probe sets (TaqMan Gene Expression Assay; Applied Biosystems, CA, USA) to measure mRNA levels of cytokines. TaqMan Gene Expression Assays used in this study included IL-4 (Mm 00445259_m1), IL-5 (Mm 000439646_m1), IL-10 (Mm 01288386_m1), IL-13 (Mm 00434204_m1), TNF-α (Mm 00443258_m1), IFN-γ (Mm 01168134_m1), and COX-2 (Mm00516005_m1).

Expression level of GAPDH (glyceraldehyde-3-phosphate dehydrogenase; Mm 99999915_g1) was used an internal control for normalization. Relative gene expression was calculated by using comparative C_T_ method (△△C_T_) with RQ Manager software 1.21 (Applied Biosystems).

After the first experiment, we found that there was no significant difference in symptom scores, ELISA, or RT-PCR results between group A and group B. In addition, we found that the therapeutic effect was more pronounced at onion extract amount of 40 μL than that at amount of 20 μL (Fig. 1). Therefore, eight mice were further assigned to groups A, C, and E for the second experiment.

### Statistical analysis

To compare results between groups, statistical analysis was carried out using ANOVA and Tukey test for normally distributed data (sneezing score and total IgE) and Kruskal-Wallis test followed by post-hoc test using Mann-Whitney U test with Bonferroni’s correction was performed for remaining values which did not follow the normal distribution (OVA-specific IgE, IL-4, IL-5, IL-10, IL-13, TNF-α, IFN- γ, COX-2 and eosinophil count). All statistical analyses were performed using PASW Statistics software ver. 18.0 (SPSS Inc., Chicago, IL, USA). A *p*-value < 0.05 was considered statistically significant.

### Ethics approval and consent to participate

Animal experiment protocol was approved by Institutional Animal Care and Use Committee (IACUC) of Samsung Biomedical Research Institute, Seoul, Korea (IACUC number: 20170117001).

## Data Availability

Data are available from the institutional animal care and use committee of samsung biomedical research institute for researchers who meet the criteria for access to confidential data.
